# Deep learning identifies antigenic determinants of severe SARS-CoV-2 infection within T-cell repertoires

**DOI:** 10.1038/s41598-021-93608-8

**Published:** 2021-07-12

**Authors:** John-William Sidhom, Alexander S. Baras

**Affiliations:** 1 Bloomberg Kimmel Institute for Cancer Immunotherapy, Johns Hopkins University School of Medicine, Baltimore, MD 21205 USA; 2 The Sidney Kimmel Comprehensive Cancer Center, Johns Hopkins University School of Medicine, Baltimore, MD 21205 USA; 3 Department of Biomedical Engineering, Johns Hopkins University School of Medicine, Baltimore, MD 21205 USA; 4 Department of Pathology, Johns Hopkins University School of Medicine, Baltimore, MD 21205 USA

**Keywords:** Computational models, Genome informatics, Machine learning, Computational biology and bioinformatics, Immunology, Biomarkers, Diseases, Medical research

## Abstract

SARS-CoV-2 infection is characterized by a highly variable clinical course with patients experiencing asymptomatic infection all the way to requiring critical care support. This variation in clinical course has led physicians and scientists to study factors that may predispose certain individuals to more severe clinical presentations in hopes of either identifying these individuals early in their illness or improving their medical management. We sought to understand immunogenomic differences that may result in varied clinical outcomes through analysis of T-cell receptor sequencing (TCR-Seq) data in the open access ImmuneCODE database. We identified two cohorts within the database that had clinical outcomes data reflecting severity of illness and utilized DeepTCR, a multiple-instance deep learning repertoire classifier, to predict patients with severe SARS-CoV-2 infection from their repertoire sequencing. We demonstrate that patients with severe infection have repertoires with higher T-cell responses associated with SARS-CoV-2 epitopes and identify the epitopes that result in these responses. Our results provide evidence that the highly variable clinical course seen in SARS-CoV-2 infection is associated to certain antigen-specific responses.

## Introduction

In December 2019, SARS-CoV-2, a novel coronavirus was first reported in Wuhan, China, and was later named a pandemic by the WHO in March 2020. Notably, the clinical course of SARS-CoV-2 infection is highly variable with individuals exhibiting asymptomatic infection all the way through to requiring critical care support in the intensive care unit (ICU)^1^. One of the defining features of SARS-CoV-2 infection that predisposes patients to requiring critical care support is acute respiratory distress syndrome (ARDS) that often complicates the clinical course of many patients; thought to be secondary to an overactive and dysfunctional/dysregulated immune response^2–8^.

Given the extent of the morbidity and mortality that has been caused by SARS-CoV-2, there has been massive efforts across academia and industry to expedite our knowledge of the disease and its corresponding immune response to develop efficacious therapies and vaccines^9–18^. In this effort, Adaptive Biotechnologies and Microsoft have partnered together to release ImmuneCODE^19,20^, a database of T-cell receptor (TCR) repertoires from individuals who were either sampled while acutely infected or recovered from SARS-CoV-2 along with derived specificities to SARS-CoV-2 epitopes through the use of the MIRA assay (Multiplex Identification of T cell Receptor Antigen Specificity)^21^.

While previous studies have focused on the transcriptional and cell profiling differences in severe infection, little work has been done to understand the antigenic determinants of clinical outcomes^14,22–25^. In the study, we sought to answer questions about the immunogenomic factors related to these antigenic determinants that may influence whether an individual develops severe versus mild SARS-CoV-2 infection by studying the T-cell repertoire in individuals during acute infection. By using DeepTCR, a deep learning software/framework for analyzing TCR-Seq^26^, to analyze the previously published and open access ImmuneCODE database, we show a TCR sequence concept signature (i.e. motif usage) that predicts severe versus mild SARS-CoV-2 infection. This work further provides a framework and example of the future use of artificial intelligence and machine learning for immune repertoire diagnostics and prognostics.

## Results

To determine the appropriate data sets to query an immune repertoire signature of severe SARS-CoV-2 infection, we carefully curated the available data within the ImmuneCODE database published by Adaptive Biotechnologies^19,20^. In the original database, there are 7 cohorts of patients or individuals who either were acutely infected or were sampled in the convalescent phase. Of the 7 cohorts, we noted that 5 (COVID-19-DLS, COVID-19-ISB, COVID-19-NIH/NIAID, COVID-19-HUniv12Oct, COVID-19-IRST/AUSL) contained patients who were sampled during active infection. Of these, 3 had clinical outcomes data that pertained to severity of illness (COVID-19-NIH/NIAID, COVID-19-ISB, COVID-19-HUniv12Oct). In the COVID-19-ISB cohort, severity of illness was measured via the WHO ordinal scale which grades degree of clinical intervention required for a given patient. In the COVID-19-NIH/NIAID and COVID-10-HUniv12Oct cohorts, there exists a label of whether the patient sampled required an intensive care unit (ICU) admission. However, when examining the proximity of sampling to active infection in the COVID-19-HUniv12Oct cohort, we noted that the majority of individuals admitted to the ICU were sampled after recovery and not during active infection (Supplementary Fig. 1). Therefore, we were left to analyze metrics of disease severity in the COVID-19-NIH/NIAID and COVID-19-ISB cohorts (here on forward referred to as NIH/NIAID and ISB respectively.

### Demographics of ImmuneCODE database

The ISB cohort was collected under the INCOVE project at Providence St. Joseph Health in Seattle, WA while NIH/NIAID was collected in Brescia, Monza and Pavia (Italy) during active infection, and provided to the NIAID in Bethesda, MD for DNA extraction. Since these two cohorts came from different parts of the world and institutions, we first wanted to examine how the demographics of the individuals in the two cohorts compared (Table 1). We noted, unsurprisingly, that these cohorts differed in composition notably in biological sex and racial groups; however, were similar in terms of age, days from symptom onset to sampling, and fraction requiring ICU-level care, as defined by the *icu_admit* label within the ImmuneCODE database or the WHO ordinal scale. Notably, we binarized the WHO ordinal scale at the point in the scale corresponding to critical care need (> 4)^27^.

**Table 1:** Demographic data of the ImmuneCODE database.

Characteristic	NIH/NIAID (N$=$ 357)	ISB (N $=$ 157)
Median age (IQR)—yr	62 (54-75)	61 (48-75)
**Biological sex**		
Male sex—no. (%)	140 (77)	71 (46)
Female sex—no. (%)	41 (23)	83 (54)
**Race—no. (%)**		
Caucasian	93 (61)	180 (100)
Asian or Pacific Islander	25 (16)	
Unknown racial group	24 (16)	
Black or African American	8(5)	
Mixed racial group	2(1)	
Days from onset to sample (IQR)—days	18 (13-25)	13 (9-21)
**Severity of illness—no. (%)**		
Severe	38 (22)	38 (36)
Mild	131 (78)	68 (64)

Demographic data for ImmuneCODE database were collected including biological sex, age, and racial group. Additionally shown, time between symptom onset and sampling along with proportion of individuals with severe illness; documented with either ICU admission or WHO ordinal scale > 4 (corresponding to individuals requiring critical care needs).

### TCR measures of diversity

**Figure 1:**
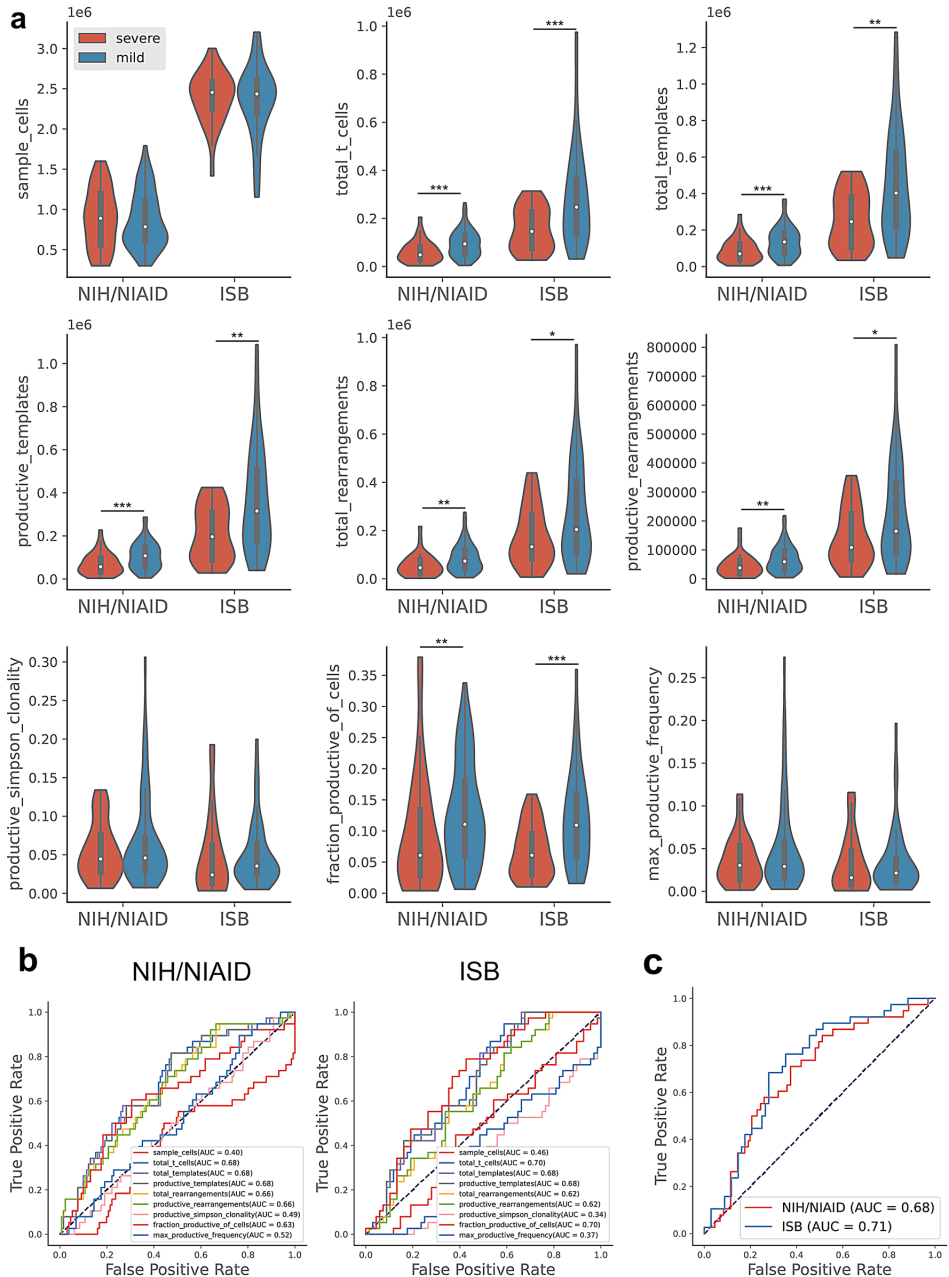
Associations of TCR diversity and abundance metrics to disease severity. (**a**) TCR-Seq diversity and abundance metrics were collected and stratified by disease severity in both the COVID-19-ISB and COVID-19-NIH/NIAID cohorts. (Mann–Whitney rank test: ***p val < 0.001, **p val < 0.01, *p val < 0.05, with multiple hypothesis testing with Benjamini/Hochberg correction, $\alpha$ = 0.05). (**b**) Uni-variate logistic regression models were fit on all TCR-Seq sample-level measures and performance was assessed via receiving operating characteristic (ROC) curves and calculating area under the curve (AUC) with 2-fold cross-validation with 100 iterations, averaging predictions across all iterations and folds. (**c**) Multi-variate logistic regression models were fit on all TCR-Seq sample-level measures and performance was assessed in the same manner as previously described in (**b**).

We first wanted to investigate whether conventional measures of TCR repertoire diversity and abundances were associated with severity of SARS-CoV-2 infection. To do this, we collected sample-level TCR-seq data provided by Adaptive Biotechnologies within the ImmuneCODE database. We first noted when looking at these metrics, that many of them were significantly different in the two cohorts (Supplementary Fig. 2), suggesting a possible difference in collection and processing of the samples prior to the sequencing. Regardless of these batch effects, when comparing all provided metrics between severe and mild infection (Fig. 1a), we noted that many of the metrics representative of a magnitude of T-cell response (i.e. total t-cells, total templates, and total rearrangements) were lower in severe versus mild infection across both the NIH/NIAID and ISB and cohorts.

To interrogate the predictive power of these associations, we fit uni-variate logistic regression on these metrics to predict severe disease and assessed performance in cross-validation (Fig. 1b). We noted that in both cohorts, a few of these associations had moderate predictive power. Finally, we fit a multi-variate logistic regression using all provided metrics, once again assessing performance in cross-validation, and found a moderate predictive power to identify individuals who had severe SARS-CoV-2 infection (Fig. 1c). Taken together, these results suggest that during the peak of infection, individuals with depleted T-cell responses have a more severe course of disease, consistent with previously described phenomena of lymphopenia in severe SARS-CoV-2 infection^28–31^.

### Deep learning models

While analyzing TCR metrics such as the abundance of T-cells, unique CDR3 sequences, or entropy can provide an assessment of the diversity of the repertoire or its level of clonal expansion, these metrics are sequence agnostic. They are unable to query the antigen-specific nature of the repertoire, and thus, cannot identify antigenic associations with clinical outcomes in this data. Therefore, we sought to use DeepTCR, a deep learning framework for analyzing TCR-Seq data, to identify antigenic determinants or TCR signatures of severe SARS-CoV-2 infection.

**Figure 2:**
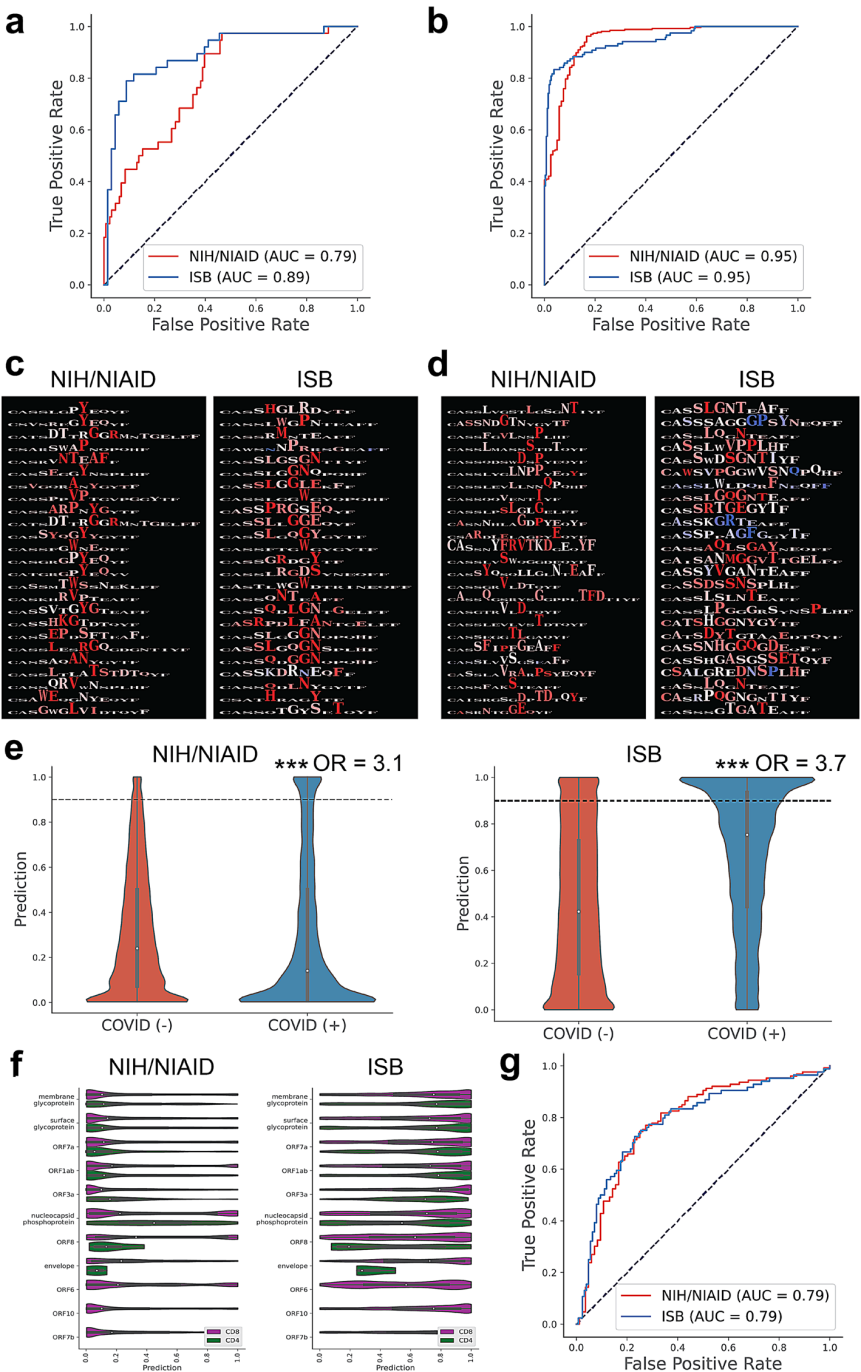
Deep learning models identify TCR signature of severe disease. (**a**) DeepTCR’s multiple instance repertoire classifier was used to fit predictive models of severe/mild illness in Monte Carlo cross-validation across both the NIH/NIAID and ISB cohort of patients. Receiver Operating Characteristic (ROC) curves are shown with corresponding Area Under Curve (AUC) measurements. (**b**) DeepTCR’s repertoire classifier was also fit to identify TCR repertoire differences between samples taken from the NIH/NIAID or ISB cohort. Receiver Operating Characteristic (ROC) curves are shown with corresponding Area Under Curve (AUC) measurements. (**c**) Following model fitting, top predictive sequences for severe disease were extracted from the network and residue sensitivity logos (RSL’s) were created highlighting predictive residues. (**d**) Following model fitting, top predictive sequences for NIH/NIAID versus ISB cohorts were extracted from the network and residue sensitivity logos (RSL’s) were created highlighting predictive residues. (**e**) All TCR sequences present in the samples were mapped to being COVID(+) or COVID(−), based on empirically derived antigen-specificity data from the MIRA assay and plotted by their corresponding prediction values for severe illness. A threshold of P = 0.90 was used to create contingency tables of TCR sequences called to carry the severe disease as well as the COVID(+) signature and used to calculate enrichment scores (Fisher’s Exact Text: ***p val < 0.001). (**f**) COVID(+) sequences were further stratified by open reading frame (ORF) in the viral genome as well as by CD8/CD4 specific TCRs as provided by the MIRA assay. (**g**) SARS-CoV-2 specific TCR sequences, as determined from MIRA assay results in the ImmuneCODE database were collected and mapped to their corresponding epitope sequences and used in place to train the repertoire classifier. Receiver Operating Characteristic (ROC) curves are shown with corresponding Area Under Curve (AUC) measurements.

In order to identify differences in the TCR repertoires of individuals who had a severe clinical course from those who had more mild symptoms, we used DeepTCR’s multiple instance repertoire classifier to fit a predictive model to the NIH/NIAID and ISB cohorts of individuals where the label of interest was severe disease either corresponding to an ICU admission in the NIH/NIAID cohort or a WHO ordinal scale score > 4 in the ISB cohort. We noted in internal Monte Carlo cross-validation, both cohorts carried significant predictive signatures (Fig. 2a. AUC = 0.79—NIH/NIAID, 0.89—ISB), which were more predictive than the logistic regression models fit to TCR metrics of abundance and diversity (Fig. 1c). However, when these models were used to predict severity of disease across cohorts, there was no shared predictive signature between them (Supplementary Fig. 3). Upon further interrogation, when attempting to fit a model to distinguish the two cohorts (NIH/NIAID and ISB) from each other, we noted a very strong predictive signature (Fig. 2b), suggesting these patients had TCR repertoires that were very different from each other and might explain why predictive models trained to predict severity of disease may not generalize across cohorts. Additionally, when combining the per-sample/patient predictions from the previously fit logistic regression model with the predictions from DeepTCR into one multi-variate logistic regression model, we noted that while there was no significant difference in performance over DeepTCR (Supplementary Fig. 4a), the two models provided independent information as evidenced by the coefficients of the logistic regression model (Supplementary Fig. 4b).

To provide more descriptive and explainable results from the deep learning models, we examined the most predictive TCR sequences and interrogated their corresponding antigen-specificities. First, we collected the top 25 most predictive sequences for severe infection and visualized them across both cohorts with residue sensitivity logos (RSL’s) from DeepTCR (Fig. 2c). As described in more detail in the original DeepTCR publication, the RSL’s query the sensitivity of any given residue to the probability of a given TCR sequence belonging to a specific class. By altering each residue, one can query the effect of this alteration on the probability output from the model and thus, determine which residues are the most relevant to the model’s prediction. We noted that the most sensitive residues were located in the central part of the CDR3 sequences, suggesting the signature was tied to an antigen-specific exposure. Furthermore, when examining the RSL’s from the model trained to distinguish between the two cohorts, we noticed that once again, the model’s attention was at the center of the CDR3 sequence, suggesting an antigen-specific difference in the cohorts (Fig. 2d).

To examine further the antigenic specificities of these TCR sequences, we used provided SARS-CoV-2 specific TCR sequences collected from the MIRA assay^21^ and provided within the ImmuneCODE database to label all TCR sequences within both cohorts by whether they were known to be COVID(+) or COVID(-) sequences. We then plotted these sequences by their prediction values to be associated with severe infection across both cohorts of patients (Fig. 2e). We noted that that when setting a threshold of *P(severe infection)* = 0.90, there was a statistically significant enrichment for SARS-CoV-2 specific TCRs in patients with severe infection. Finally, when stratifying the SARS-CoV-2 positive TCRs by open reading frame (ORF) and by CD8 or CD4 specificity, we noted in both cohorts that certain areas of the viral genome had increased likelihood of being targeted in severe infection as well as there also being differences in the CD8 versus CD4 response across multiple ORFs (Fig. 2f). Full results have been provided in Supplementary Tables 1 and 2 allowing investigators to query the most immunodominant/immunogenic epitopes and their corresponding TCRs.

Finally, in order to interrogate further what this difference in antigen-specificity could be within the two cohorts analyzed, we used provided the SARS-CoV-2 specific TCR sequences collected from the MIRA assay to map TCR sequence to known SARS-CoV-2 epitopes and when substituted these sequences into DeepTCR’s repertoire classifier to query whether there was a similar ability for the model now fit to the epitope sequence to predict the cohort a given sample belonged to. In this case, the input sequencing data was the epitope sequence data for the SARS-CoV-2 specific TCRs (as determined by MIRA), and the predicted label was the cohort the given sample came from. We found that while not to the same predictive power as when the model was fit to TCR sequences, there still remained the ability for DeepTCR’s repertoire classifier to predict the cohort label from the “epitope repertoire” (Fig. 2g), suggesting once again the SARS-CoV-2 specific response was antigenically different between these two cohorts. Taken together, these results reveal an antigen-specific signature associated with SARS-CoV-2 specific epitopes that is predictive of severe clinical course in SARS-CoV-2 infection and furthermore, the SARS-CoV-2 specific repertoire recognizes different epitopes depending on the population sampled.

## Discussion

In this work, we demonstrate the power of leveraging a multiple instance deep learning framework within a clinically relevant and timely cohort of individuals to predict and learn distinguishing TCR sequence features in individuals with clinically severe SARS-CoV-2 infection. We further identify that this predictive signature is associated with SARS-CoV-2 specific TCRs as determined by MIRA, an orthogonal T-cell culture based sequencing assay and also reveal that the antigenic response may be dependent on the population sampled. We believe this work highlights multiple points of novelty for physicians and scientists studying SARS-CoV-2 but also for the broader scientific community interested in leveraging immune repertoire for diagnostic and prognostic purposes.

A few limitations exist in this study that are important when considering the interpretations and conclusions of this work. The first major limitation we found as was highlighted in the cross-validation of the models between cohorts was the predictive signature was not shared between the NIH/NIAID and ISB cohorts. We noted that there are multiple reasons this could be the case. The first reason one must consider when using machine learning models is over-fitting of the model. While this could be playing a role in why the models did not cross-validate, there are several other notable reasons that might explain the lack of generalization. First, as was described in the demographics of the cohorts, these two cohorts were notably different in biological sex and race. Previous studies have revealed differences in clinical outcomes as a function of sex and race^32–36^ and therefore; these differences could be playing a large role in why the models do not generalize across cohorts. Second, when looking at the measures of TCR abundance and diversity between the two cohorts, there were clear batch effects that may reflect differences in how the samples were collected and processed. These artifactual differences could also add to the difficulty of the model generalizing between cohorts. Furthermore, when we trained a repertoire classifier to distinguish between samples from either cohort, the model had very high power in distinguishing the repertoires from individuals that came from either cohort, further providing evidence for batch effects in the sequencing data. Whether these batch effects were derived from biological sources of variation (i.e. immunogenetic background, environmental exposures, etc) or technical sources of variation (i.e. collection protocol, sample preparation, sequencing differences, etc), we cannot be sure of but the fact that a repertoire model trained on the corresponding “epitope repertoire” also provided predictive power to distinguish the cohorts suggests a relevant biological difference between the cohorts. Furthermore, when looking at the SARS-CoV-2 antigenic signature in both cohorts as it related to the predictive signature of clinical severity, we noted a stronger association between the predictive signature in the ISB to the antigenic signature than in the NIH/NIAID cohort. One possible explanation of this is due to the cohort of patients the MIRA assay was performed. If the MIRA assay patients were collected in the USA, the SARS-CoV-2 response may be different in this demographic versus the Mediterranean Italian cohort in the NIH/NIAID cohort. Ideally, one would have MIRA data matched for the demographics of the cohort one was training the repertoire models on. These results should encourage investigators in the future to study the host-specific factors that could shape the antigen-specific immune response in patients with not only just SARS-CoV-2 but all other insults whether they be pathogenic, autoimmune, or oncological in origin.

Another significant limitation, particularly of the ImmuneCODE database is the clinical outcomes data associated with the repertoire data. As was noted, not only do two of the five cohorts with patients who were sampled while acutely infected lack clinical outcomes data, the HUniv12Oct cohort had collected outcomes data at different time points with respect to the peak of infection. Given the nature of how immune responses evolves over the course of infection, further studies should provide more descriptive annotations of when samples were collected with respect to symptom onset, if/when a patient was admitted to the ICU, and if/when they recovered. Furthermore, sampling from multiple time points over the course of illness would also help distinguish host-specific versus disease specific features of the immune repertoire as they relate to clinical outcomes. Since the cohorts of patients we studied here were only sequenced during the acute phase of their infection, one cannot determine if the predictive features were disease of host-specific.

In conclusion, SARS-CoV-2 represents an interesting scientific and clinical phenomena where a single pathogen can cause a highly variable clinical presentation, which has led scientists to look for immune related factors that may explain this variable presentation. Here, by applying deep learning of the immune repertoire, we reveal a TCR signature that is predictive of severe versus mild infection, which not only provides insight into the immune response to SARS-CoV-2 but also provides a framework to study host-specific differences in immune repertoire as they relate to clinically relevant outcomes.

## Methods

### Data collection and curation

All data used in this study was collected from the publicly available ImmuneCODE Open Access Database^19,20^. Data was pre-processed and organized to be compatible with DeepTCR. Steps to pre-process and organize data can be found in scripts in the code repository (referenced under Code availability). The data in this study consists of 1) clinical metadata, 2) TCR repertoire data, and 3) antigen-specific TCR data collected via the MIRA assay^21^. Severity of illness was determined either by whether the individual had an ICU admission (NIH/NIAID) or whether their WHO ordinal scale was greater than 4 (ISB).

### Logistic regression models

Logistic regression models were fit to predict severity of SARS-CoV-2 infection from aggregate TCR metrics provided under the ImmuneCODE database. Models were implemented with scikit-learn python package and performance was assessed via 2-fold cross-validation with 100 iterations to obtain per-sample prediction. Performance was assessed with Receiver Operating Characteristic (ROC) curves and Area Under Curve (AUC) measurements.

### Training deep learning repertoire classifier

DeepTCR’s multiple instance repertoire classifier was used to fit predictive models based on the TCR repertoires of individuals who were acutely infected with SARS-CoV-2 and had documented clinical outcomes in terms of severity of infection. Models were fit in Monte Carlo cross-validation fashion and per-sample predictions were averaged over all Monte Carlo simulations. All DeepTCR hyper-parameters can be found under training scripts within the code repository (referenced under Code availability). Performance was assessed with ROC curves and AUC measurements.

### Statistical tests and machine learning models

All statistical tests applied to data were implemented with the scipy.stats module. Classical machine learning techniques and performance metrics were implemented with scikit-learn. Deep learning models were implemented with DeepTCR (v2.0.10) python package (https://pypi.org/project/DeepTCR/)^26^.

## Supplementary Information

Supplementary Figures.

Supplementary Tables.

## Data Availability

All data used in this study is publicly available as part of the ImmuneCODE Open Access Database^19,20^ and available for download at https://clients.adaptivebiotech.com/pub/covid-2020.

## Code availability

All code used to analyze this dataset can be found at https://github.com/sidhomj/DeepTCR_COVID19.

## References

[1] Goyal P (2020). Clinical characteristics of Covid-19 in New York city. N. Engl. J. Med..

[2] Yao C (2021). Cell-type-specific immune dysregulation in severely ill Covid-19 patients. Cell Rep..

[3] Kalfaoglu B, Almeida-Santos J, Tye CA, Satou Y, Ono M (2021). T-cell dysregulation in Covid-19. Biochem. Biophys. Res. Commun..

[4] Acharya D, Liu G, Gack MU (2020). Dysregulation of type I interferon responses in Covid-19. Nat. Rev. Immunol..

[5] Qin C (2020). Dysregulation of immune response in patients with coronavirus 2019 (Covid-19) in Wuhan, China. Clin. Infect. Dis..

[6] Zheng M (2020). Functional exhaustion of antiviral lymphocytes in Covid-19 patients. Cell. Mol. Immunol..

[7] Li X, Geng M, Peng Y, Meng L, Lu S (2020). Molecular immune pathogenesis and diagnosis of Covid-19. J. Pharm. Anal..

[8] Shi Y, Wang Y, Shao C (2020). COVID-19 infection: the perspectives on immune responses. Cell Death Differ..

[9] Chevrier S (2021). A distinct innate immune signature marks progression from mild to severe Covid-19. Cell Rep. Med..

[10] Abers MS (2021). An immune-based biomarker signature is associated with mortality in Covid-19 patients. JCI Insight.

[11] d’Alessandro M (2021). Peripheral biomarkers’ panel for severe Covid-19 patients. J. Med. Virol..

[12] Liao M (2020). Single-cell landscape of bronchoalveolar immune cells in patients with Covid-19. Nat. Med..

[13] Zhang B (2021). The dynamics of immune response in Covid-19 patients with different illness severity. J. Med. Virol..

[14] Hansen CB (2021). SARS-CoV-2 antibody responses are correlated to disease severity in Covid-19 convalescent individuals. J. Immunol..

[15] Kusnadi A (2021). Severely ill Covid-19 patients display impaired exhaustion features in SARS-CoV-2-reactive CD8+ T cells. Sci. Immunol..

[16] Kang CK (2020). Aberrant hyperactivation of cytotoxic T-cell as a potential determinant of Covid-19 severity. Int. J. Infect. Dis..

[17] Diao B (2020). Reduction and functional exhaustion of T cells in patients with coronavirus disease 2019 (Covid-19). Front. Immunol..

[18] Kalfaoglu B, Almeida-Santos J, Tye CA, Satou Y, Ono M (2020). T-cell hyperactivation and paralysis in severe Covid-19 infection revealed by single-cell analysis. Front. Immunol..

[19] Dines, J. N. *et al.* The immunerace study: A prospective multicohort study of immune response action to Covid-19 events with the immunecode open access database. *medRxiv* (2020).

[20] Nolan, S. *et al.* A large-scale database of T-cell receptor beta (TCR) sequences and binding associations from natural and synthetic exposure to SARS-CoV-2. *Res. Sq.* (2020). 10.3389/fimmu.2025.1488851 PMC11873104 40034696

[21] Klinger M (2015). Multiplex identification of antigen-specific T cell receptors using a combination of immune assays and immune receptor sequencing. PLoS One.

[22] Kroemer M (2021). Covid-19 patients display distinct SARS-CoV-2 specific T-cell responses according to disease severity. J. Infect..

[23] Sattler A (2020). SARS-CoV-2-specific T cell responses and correlations with Covid-19 patient predisposition. J. Clin. Investig..

[24] Bacher P (2020). Low-avidity CD4+ T cell responses to SARS-CoV-2 in unexposed individuals and humans with severe Covid-19. Immunity.

[25] Dykema, A. G. *et al.* Functional characterization of CD4+ T-cell receptors cross-reactive for SARS-CoV-2 and endemic coronaviruses. *J. Clin. Investig.* (2021). 10.1172/JCI146922 PMC8121515 33830946

[26] Sidhom J-W, Larman HB, Pardoll DM, Baras AS (2021). DeepTCR is a deep learning framework for revealing sequence concepts within T-cell repertoires. Nat. Commun..

[27] McCreary EK, Angus DC (2020). Efficacy of remdesivir in Covid-19. Jama.

[28] Li T (2004). Significant changes of peripheral T lymphocyte subsets in patients with severe acute respiratory syndrome. J. Infect. Dis..

[29] Lippi G, Plebani M (2020). Laboratory abnormalities in patients with Covid-2019 infection. Clin. Chem. Lab. Med. (CCLM).

[30] Tan L (2020). Lymphopenia predicts disease severity of Covid-19: A descriptive and predictive study. Signal Transduct. Target. Therapy.

[31] Huang I, Pranata R (2020). Lymphopenia in severe coronavirus disease-2019 (Covid-19): Systematic review and meta-analysis. J. Intensive Care.

[32] Apea VJ (2021). Ethnicity and outcomes in patients hospitalised with Covid-19 infection in east London: An observational cohort study. BMJ Open.

[33] Ebinger JE (2020). Pre-existing traits associated with Covid-19 illness severity. PLoS One.

[34] Zakeri R (2020). A case-control and cohort study to determine the relationship between ethnic background and severe Covid-19. EClinicalMedicine.

[35] Meng Y (2020). Sex-specific clinical characteristics and prognosis of coronavirus disease-19 infection in Wuhan, China: A retrospective study of 168 severe patients. PLoS Pathog..

[36] Bunders, M. J. & Altfeld, M. Implications of sex differences in immunity for SARS-CoV-2 pathogenesis and design of therapeutic interventions. *Immunity* (2020). 10.1016/j.immuni.2020.08.003 PMC7430299 32853545

