# Supplementary material for: Deep learning identifies antigenic determinants of severe SARS-CoV-2 infection within T-cell repertoires
Source: Sci Rep. 2021 Jul 12;11:14275. doi: 10.1038/s41598-021-93608-8 (PMC8275616; doi:10.1038/s41598-021-93608-8)
Supplement: Supplementary file 1 — Supplementary Figures. [file 41598_2021_93608_MOESM1_ESM.pdf]

Supplementary Figures

1. **Days from Recovery to Sample in COVID-19-HUniv12Oct data set**
2. **TCR Diversity Metrics of ISB & NIH/NIAID Cohorts**
3. **Cohort Cross-Validation**
4. **Multi-variate logistic regression using DeepTCR and TCR metrics of abundance and diversity**

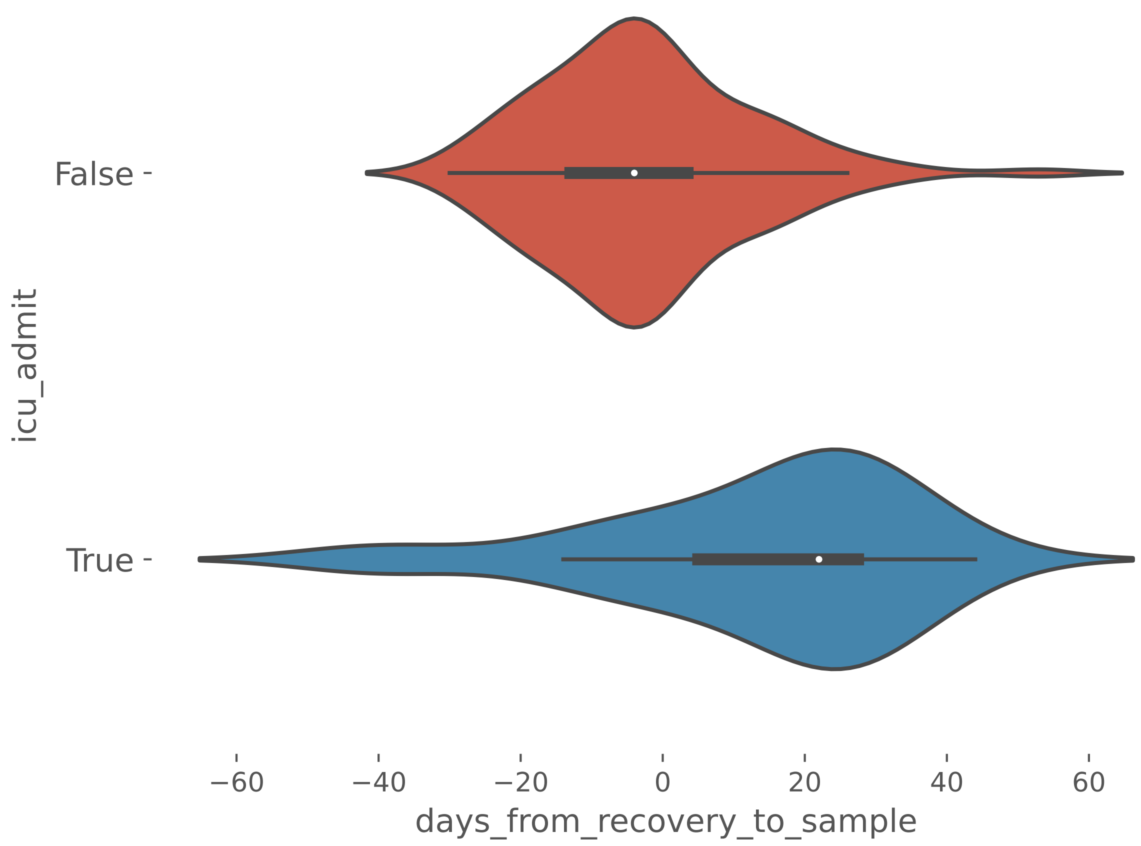

Supplementary Figure 1. Days from Sample to Recovery in COVID-19-HUniv12Oct data set Sample metadata was collected to visualize number of days from recovery to sample, stratified by whether the patient was admitted to the intensive care unit (ICU).

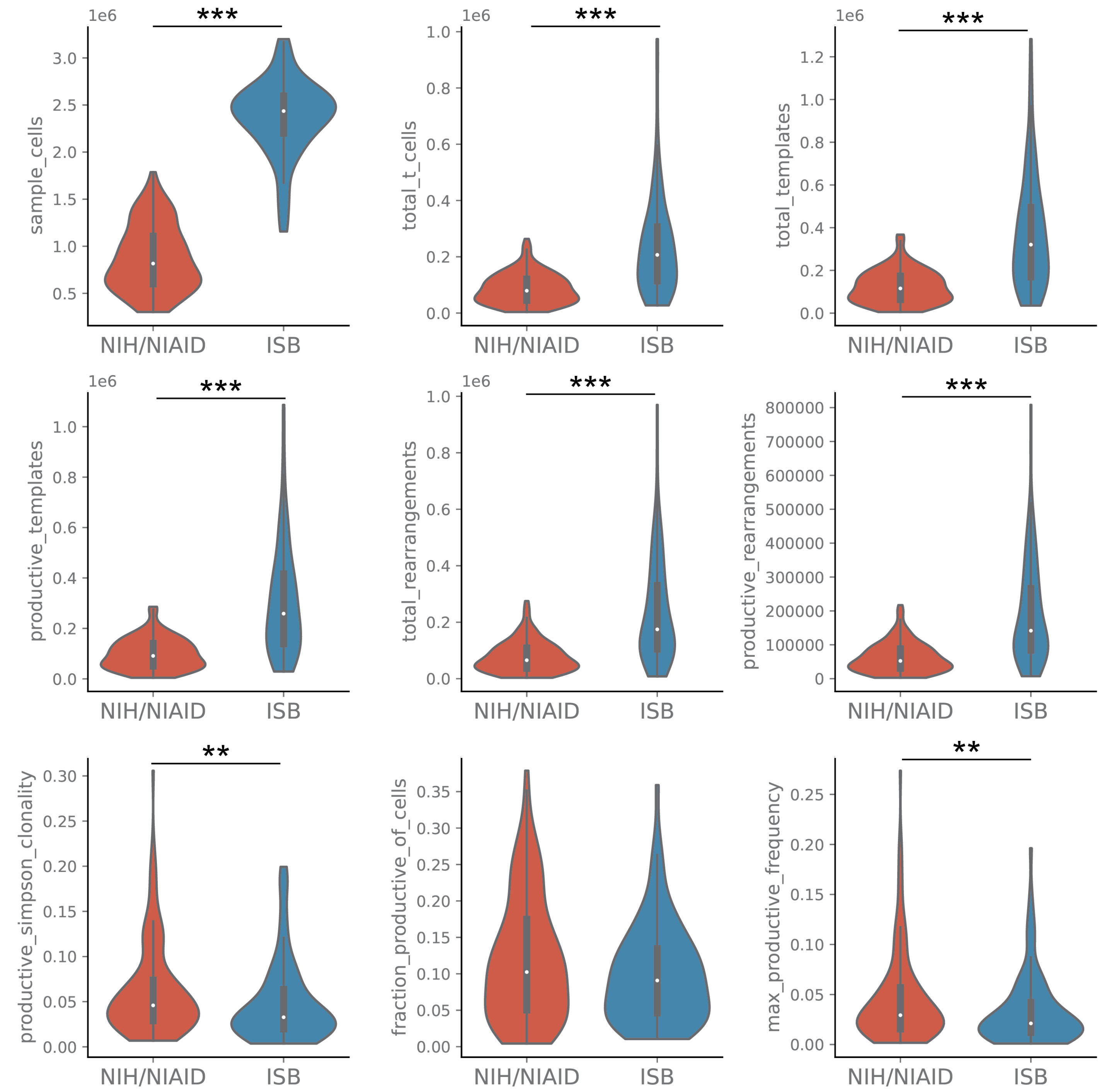

Supplementary Figure 2. TCR Diversity Metrics of ISB & NIH/NIAID Cohorts Sample metadata along with TCR metrics produced in original ImmuneCODE data set were collected to visualize and analyze TCR diversity differences between the COVID-19-ISB & COVID-19-NIH/NIAID cohhorts. (Mann-Whitney rank test: *** p-val < 0.001, ** p-val < 0.01)

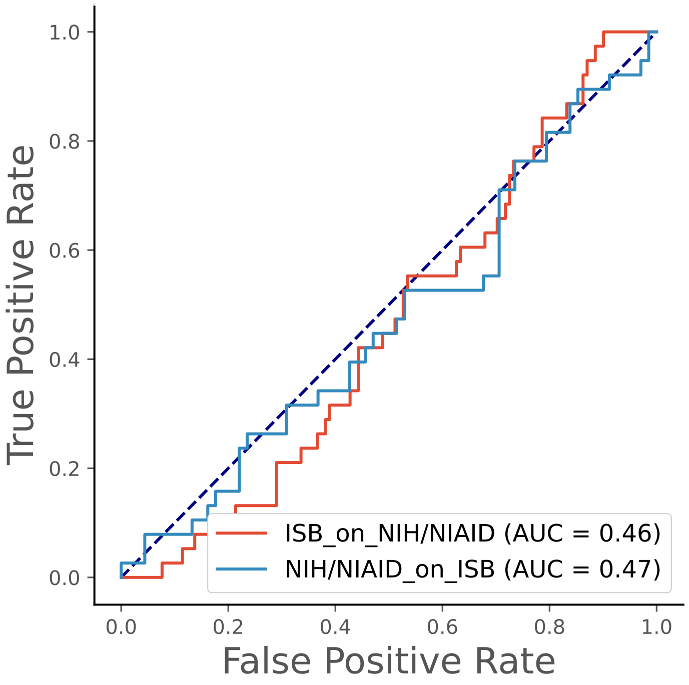

Supplementary Figure 3. Cohort Cross-Validation. DeepTCR's repertoire classifier was fit to either the NIH/NIAID or ISB cohort and then was used to conduct sample-level inference on the other cohort of patients. Receiver Operating Characteristic (ROC) curves are shown with corresponding Area Under Curve (AUC) measurements.

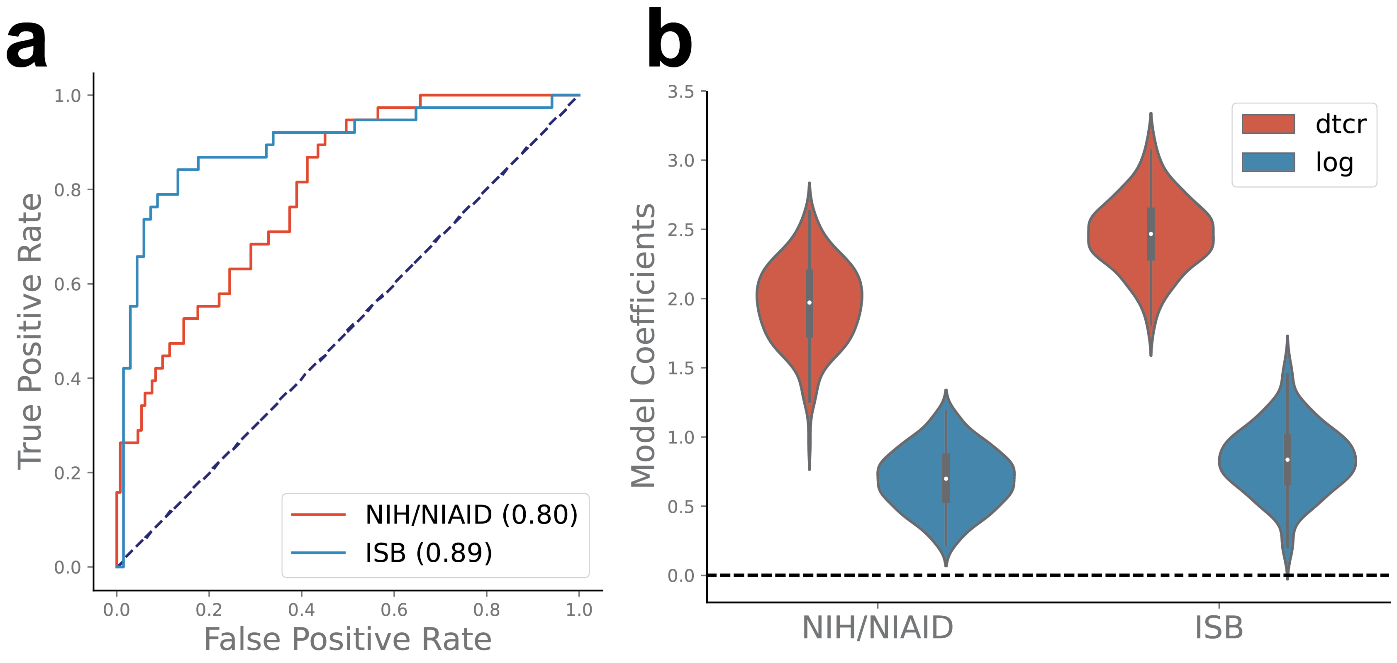

Supplementary Figure 4. Multi-variate logistic regression using DeepTCR and TCR metrics of abundance and diversity. (a) Per sample predictions from logistic regression model (Fig. 1c) and DeepTCR (Fig. 2a) were collected for both the NIH/NIAID and ISB cohorts of patients and used to fit a multi-variate logistic regression to predict severe vs. mild disease. Receiver Operating Characteristic (ROC) curves are shown with corresponding Area Under Curve (AUC) measurements. **(b)** Logistic regression coefficients were collected following fitting the model over 100 iterations and shown for both cohorts of patients. When DeepTCR and TCR metrics of abundance/diversity are used in multivariate logistic regression modeling, they remain as two independent predictors as evidenced by the fact that the 99% bootstrapped confidence intervals of the model coefficients do not cross 0 (NIH/NIAID 99% CI; DeepTCR = [1.308, 2.561], TCR metrics = [0.182, 1.228]: ISB 99% CI; DeepTCR = [1.800, 3.091], TCR metrics = [0.255, 1.430])
